# Plasmonic Gold Nanostars for Multi-Modality Sensing and Diagnostics

**DOI:** 10.3390/s150203706

**Published:** 2015-02-05

**Authors:** Yang Liu, Hsiangkuo Yuan, Farrell R. Kersey, Janna K. Register, Matthew C. Parrott, Tuan Vo-Dinh

**Affiliations:** 1 Fitzpatrick Institute for Photonics, Duke University, Durham, NC 27708, USA; E-Mails: yl183@duke.edu (Y.L.); hkscott1125@gmail.com (H.Y.); janna.register@duke.edu (J.K.R.); 2 Department of Biomedical Engineering, Duke University, Durham, NC 27708, USA; 3 Department of Chemistry, Duke University, Durham, NC 27708, USA; 4 Department of Radiology & Biomedical Research Imaging Center, University of North Carolina at Chapel Hill, NC 27510, USA; E-Mails: fkersey@email.unc.edu (F.R.K.); parrotmc@email.unc.edu (M.C.P.)

**Keywords:** gold nanostars, plasmonics, multifunctional, sensing

## Abstract

Gold nanostars (AuNSs) are unique systems that can provide a novel multifunctional nanoplatform for molecular sensing and diagnostics. The plasmonic absorption band of AuNSs can be tuned to the near infrared spectral range, often referred to as the “tissue optical window”, where light exhibits minimal absorption and deep penetration in tissue. AuNSs have been applied for detecting disease biomarkers and for biomedical imaging using multi-modality methods including surface-enhanced Raman scattering (SERS), two-photon photoluminescence (TPL), magnetic resonance imaging (MRI), positron emission tomography (PET), and X-ray computer tomography (CT) imaging. In this paper, we provide an overview of the recent development of plasmonic AuNSs in our laboratory for biomedical applications and highlight their potential for future translational medicine as a multifunctional nanoplatform.

## 1. Introduction

Sensitive and selective detection methods have important applications for biomedical sensing and clinical diagnostics. Raman spectroscopy is a non-destructive photon-scattering technique, which provides useful spectral information related to specific molecular vibrational energy levels of the molecules being monitored [1–4]. However, the efficiency of Raman scattering is intrinsically low: even strong Raman scatterers exhibit cross-sections only on the order of 10^−29^ cm^2^ molecule^−1^ sr^−1^ while the fluorescence cross-section is usually on the order of 10^−16^ cm^2^ molecule^−1^ sr^−1^ [5]. The surface-enhanced Raman scattering (SERS) effect takes advantage of the locally enhanced electromagnetic field that occurs when light irradiates metallic nanostructures to amplify Raman scattering, resulting in a sensitive and specific method for chemical analysis [6,7]. It has advantages of high sensitivity, spatial resolution and the capability of multiplexed detection. The SERS effect was discovered in the mid-1970s [8,9] and the first analytical application of SERS in chemical analysis using nanostructured metal substrates was demonstrated in our laboratory [8–10]. We introduced a unique type of plasmonics-active substrate, a metal film on nanoparticle (MFON) structure, as efficient and reproducible SERS-active media, which has led to a wide variety of SERS nanoplatforms consisting of many shapes and structures ranging from metal film on nanoparticle-coated microplates, nanogratings, nanorod arrays, optical fibers, nanodots, nanowires and nanostars for use in chemical sensing, bioanalysis and biosensing [11–20]. Extensive research has been devoted to understanding and modeling the Raman enhancement in SERS since the mid-1980s and results suggested that the SERS effect primarily arises from electromagnetic resonance occurring near metallic nanostructure surface [21–25]. When the metallic nanostructure surface is irradiated by an external electromagnetic field, electrons within the conduction band oscillate at the same frequency. These oscillating electrons are known as surface plasmons, which produce a secondary electric field added to the external electromagnetic field, resulting in surface plasmon resonance [7,23]. The secondary electric field is typically most concentrated on the rough metallic nanostructure surface [24,26]. In addition, the SERS effect can also be due to a chemical enhancement associated with interaction between molecules and metal surface. The resulting SERS enhancement factor can be 10^6^ to 10^8^-fold, and up to 10^15^-fold at “hot spots” where electromagnetic field enhancements of multiple plasmonic nanoparticles overlap in small spaces [5,27]. With such a high sensitivity, SERS has been shown to be a powerful chemical analysis technique with sensitivity reaching the single-molecule level [28].

We have developed a novel surfactant-free method to synthesize star-shaped gold nanoparticles, gold nanostars (AuNSs), which have multiple sharp branches creating “lightning rod” effect that enhances the local electromagnetic field dramatically [14,29]. The developed AuNSs have a tunable plasmonic band in the near-infrared (NIR) region where tissue absorption is minimal [30]. We demonstrated that plasmon resonance significantly increases the two-photon action cross-sections (TPACS) to more than a million GM (Göeppert-Mayer units). TPACS of AuNSs is greater than that of nanorods and organic fluorophores [29]. Our group has demonstrated for the first time that AuNSs, with intense NIR contrast under multiphoton microscopy, can be visualized in real time [31]. Based on the advantages of AuNSs including a tunable NIR plasmonic band, strong electromagnetic field enhancements at branches and toxic surfactant-free synthesis, their applications have been numerous in the biomedical field, including SERS, two-photon photoluminescence (TPL), photoacoustic imaging and biosensing [26,32–37].

One particularly important biomedical application is in the area of cancer research. Cancer has become one of the leading causes of death in the world and it is estimated that there are more than 12.7 million cancer cases and 7.6 million deaths each year [38]. The developed AuNSs provide a superior nanoplatform for multimodal imaging for cancer diagnostics due to its large surface area for linking other contrast agents, like Gd^3+^ and ^64^Cu, for magnetic resonance imaging (MRI) and positron emission tomography (PET) imaging [37]. AuNSs with sizes less than 100 nm can accumulate selectively in tumors *via* the well-known enhanced permeability and retention (EPR) effect, which is due to the increased leakiness of blood vasculature in tumors [39–41]. In addition to the passive EPR mechanism, targeting ligands including antibodies and peptides, can also be chemically bonded to the AuNSs surface to perform active targeting [42,43]. Therefore, AuNSs could be used for targeted multimodal imaging for cancer diagnostics. In this article, we will review the recent progress in our laboratory on biosensing and multi-modality imaging with AuNSs for biomedical applications.

## 2. AuNSs with Tunable Plasmonic Properties

**Figure 1.**
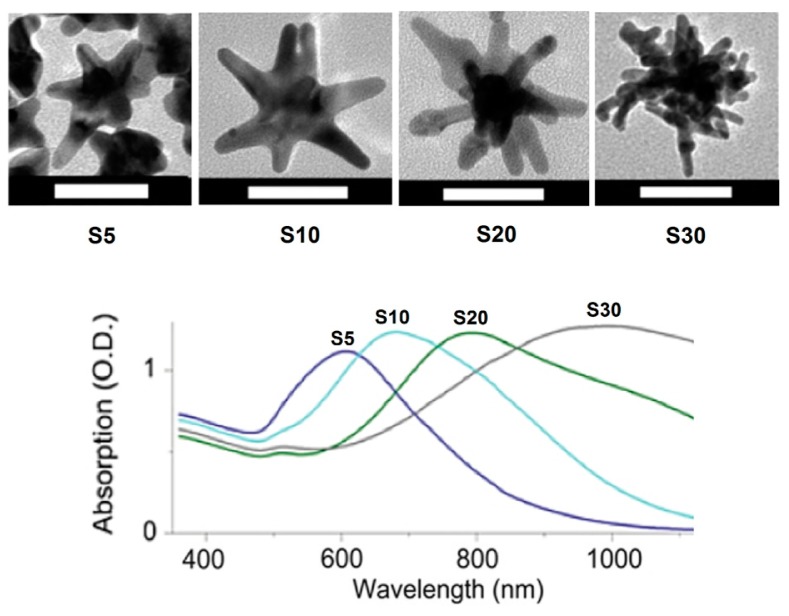
**Top**: TEM images of gold nanostars (AuNSs) formed under different Ag^+^ concentrations *via* a seed-mediated method using AgNO_3_ (S5: 5 μM, S10: 10 μM, S20: 20 μM, S30: 30 μM). The scale bar is 50 nm; **Bottom**: extinction spectra of synthesized AuNSs solutions (∼0.1 nM) in water [29]. (Adapted from Ref. [29]).

AuNSs have been synthesized with our unique seed-mediated method that does not require the use of the toxic surfactant cetrimonium bromide (CTAB), making them suitable for *in vivo* biomedical applications [29]. The plasmonic properties of the AuNSs can be modified to match the laser frequency for imaging and therapy by controlling the geometry of AuNSs with various concentrations of Ag^+^ in the synthesis process. As shown in the Figure 1, high concentration of Ag^+^ in the AuNSs synthesis can red shift the plasmonic band by forming longer, sharper and more branches. For example, the S30 AuNSs have much more branches with longer and sharper shapes than that of S5 ANSs. We also performed theoretical simulations by using the finite element model (FEM), and results show that significant localized electromagnetic field enhancement is observed on the branch tips, which is associated with “lightning rod” effect. AuNSs synthesized in our laboratory offer an important nanoplatform for light-based diagnostics and therapeutics with their unique optical properties. AuNSs have been used to develop substrate for SERS measurement [44]. The 3-aminopropyltriethoxysilane (APTES)-functionalized method was used to link AuNSs to glass slip surface to develop a reproducible SERS substrate with reported enhancement factor of 5 × 10^6^ [45]. In addition, another study mentioned applied nanolithography and electroless deposition to synthesize a homogeneous substrate with AuNSs for pH sensitive measurement with SERS [46]. Furthermore, AuNSs have also been used for tissue imaging of the tumor suppressor p63 with SERS as well as lymphatic system imaging with photoacoustic mapping [47–49].

## 3. AuNSs for pH Sensing

Solid tumors have been reported to contain volumes that are highly acidic, which are due to high rates of glucose metabolism and poor perfusion [50,51]. Fluorescence-based probes were previously used for pH sensing [52]. However, those fluorophores undergo irreversible photobleaching within seconds to minutes [53]. Furthermore, fluorescence methods cannot be applied to detect multiple biomarkers at the same time due to their broad and featureless spectrum nature. The SERS method has been investigated as an alternative approach for pH sensing [28,54,55]. Our group has developed a fiber-optic nanoprobe with a silver coating for intracellular pH sensing and the results demonstrate that the fiber-optic nanoprobe can be applied to monitor the pH environment at the single cell level [5]. In addition, silver colloid solutions have also been used to develop SERS nanoprobes for intracellular pH detection, and the pH value inside HeLa cancer cells was reported to be in the range of 4–5 for the majority of locations [55]. Although silver nanoparticles have been successfully used for pH sensing, gold is considered to be more suitable for *in vivo* applications since it is chemically inert and typically non-toxic [29,33,35,56].

**Figure 2.**
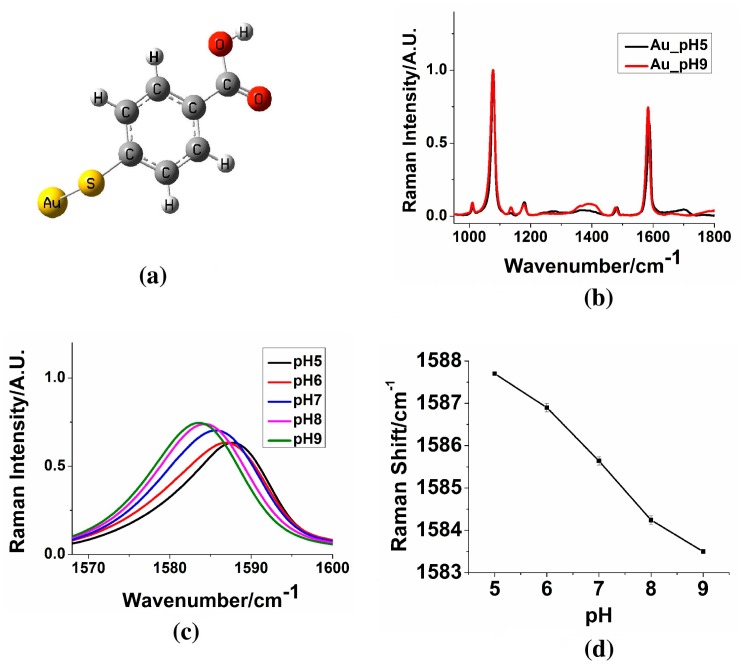
(**a**) pMBA-Au complex structure used for density functional theory (DFT) calculations; (**b**) Surface enhanced Raman scattering (SERS) spectra comparison obtained from pH sensing AuNSs nanoprobes at pH 5 and 9. The spectrum is normalized with intensity at 1078.3 cm^−1^; (**c**,**d**) SERS peak position shift between pH 5 and 9 [27]. (Adapted from Ref. [27]).

AuNSs exhibit an extremely strong SERS enhancement at their multiple branches. Previous studies have demonstrated that the SERS enhancement of AuNSs could be more than 100 times stronger than traditional gold nanospheres with similar size, making it possible to develop a gold-based pH sensing nanoprobe [4]. Our developed AuNSs nanoprobe with p-mercaptobenzoic acid (pMBA) for pH sensing shows sensitive SERS spectral changes in the pH range 5–9 (Figure 2) [27]. The peak position at ∼1580 cm^−1^ exhibits a small but noticeable blue-shift when pH changes from 5 to 9. The peak intensities at 1010.4 cm^−1^, 1136.8 cm^−1^, and 1390.1 cm^−1^ increase as the pH changes from 5 to 9. In contrast, the peak intensity at 1700.0 cm^−1^ decreases when the pH changes from 5 to 9. Theoretical simulations have been used to study molecular properties including molecular orbitals, transition states, vibrational modes and condensed phase properties [3,57–60]. We have performed density functional theory (DFT) calculations to investigate vibrational modes for the SERS peaks observed experimentally. Furthermore, the SERS spectrum changes with pH were well explained with theoretical study. Peak ratios with COOH stretching mode has been previously used for pH sensing [61]. However, the peak for COOH group stretching at 1700.0 cm^−1^ is relatively broad and weak. As a result, the calculated pH value based on peak ratios might be affected by background signal. This problem becomes more severe for *in vivo* applications since the background in biological systems is much more complicated than that in solution.

We identified a novel pH sensing index, the SERS peak shift at ∼1580 cm^−1^ experimentally and explained the observed peak shift with theoretical calculations. The peak position shift was found to be due to the vibrational mode coupling between the benzene ring and carboxylic group stretching. The SERS peak at ∼1580 cm^−1^ exhibits a much stronger intensity (∼10 times) than the peak at 1700.0 cm^−1^. In addition, the SERS peak at ∼1580 cm^−1^ is much sharper than that at 1700.0 cm^−1^. Therefore, the calculated pH based on the SERS peak at ∼1580 cm^−1^ can be anticipated to be less affected by environmental background signal and more suitable for *in vivo* applications. This is the first demonstration that the SERS peak position can be used as a pH-sensing index. The developed pH sensing nanoprobes and identified novel pH sensing index could be applied for future local cellular environment investigations and has potential for *in vivo* tumor boundary delineation.

## 4. AuNSs for Brain Tumor Imaging

Another very promising application for AuNSs is brain tumor imaging. Malignant glioma is the most common form of brain tumor and despite decades of efforts, median outcome survival (OS) still remains dismal [62]. Surgery followed by radiation and chemotherapy is the typical treatment for gliomas but while these techniques often shrink primary tumors, recurrence and progression are common leading to poor median OS. One potential explanation lies in the inefficient delivery of diagnostic or therapeutic agents to the tumors due to the presence of the blood-brain barrier (BBB) [63]. The BBB is a protective barrier that prevents drug molecules and contrast agents from entering the tumor parenchyma [64]. Therefore, it is of great interest to investigate how to overcome the BBB for brain tumor management.

Several nanoplatforms have been introduced to treat malignant brain tumors and other CNS disorders [65–67]. For instance, transferrin-containing gold nanoparticles [68], polysorbate 80-coated poly(n-butyl cyanoacrylate) dextran polymers [69] and angiopep2-functionalized dendrimers [70] have been investigated optically to demonstrate their capability to traverse the BBB. For diagnostic and therapeutic purposes, nanoparticles are generally required to survive immunoclearance, accumulate in the tumor, extravasate tumor vessels, diffuse through interstitial matrices, and enter the plasma membrane into cells [71,72]. In spite of extensive investigations, the actual brain uptake 24-hour post-injection may still be less than 0.1% of the initial dosage [73]. Even though those nanoparticles can be detected in the brain tumor in some studies, it does not necessarily imply that they accumulate in the tumor parenchyma more than in the intravascular space, perivascular space, or other organs. Biodistribution assessment on a whole brain using elemental analysis (e.g., ICP-MS) or low-resolution radioisotope imaging is sufficient for quantification but cannot distinguish intravascular or intraparenchymal accumulation; whole-body imaging evidence of tumor accumulation may simply reflect nanoparticles in the tumor vessels but not in the tumor parenchyma and hence may overestimate the tumor accumulation. Additionally, labeled dye on nanoparticles may dissociate in animals making imaging characterization error prone, hence a nanoplatform such as plasmonics-active nanoparticle with intrinsic imaging properties that resists biodegradation would be desirable. High-resolution optical imaging of plasmonic nanoprobes, which exhibit intravascular stability and high contrast mechanism, can therefore be applied to better understand their actual delivery into the brain tumor.

**Figure 3.**
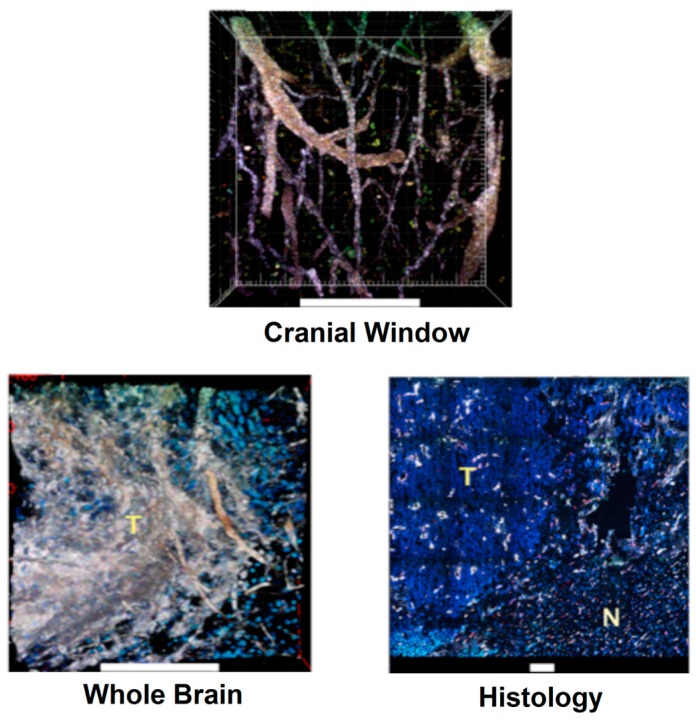
Two-photon luminescence (TPL) imaging of cerebral microangiogram, Hoescht 33342-stained whole brain, and DAPI/CD31-stained cryosectioned histology. AuNSs are white. T: tumor. N: normal. Scale bar: 200 μm [36]. (Adapted from Ref. [36]).

AuNSs, as a strong optical contrast agent with exceptionally high two-photon luminescence (TPL) signal, offer superior flexibility to investigate how drug nanocarriers and contrast agents can be delivered into brain tumors [29,35]. We employed intravital microscopy using a multi-photon microscope to examine AuNSs intratumoral distribution in the brain with high spatial resolution. Figure 3 illustrates a high-resolution depth-resolved *in vivo* cerebral microangiogram taken through a cranial window with clearly visible capillaries and minimal tissue autofluorescence background. AuNSs exhibit longer serum half-life (several hours) than that of commercial intravascular contrast agents (e.g., FITC-dextran) that undergo significant signal decay in less than 30 min. Three hours following systemic injection of AuNSs (coated with PEG polymer), AuNSs accumulated preferentially in the tumor than the surrounding normal area. Upon histological examination counterstained with DAPI, AuNSs not only accumulated in the tumor vascular endothelial cells (ECs), but also selectively penetrated the tumor BBB. Passive accumulation of AuNSs to the tumor periphery is most likely due to the hyper-neovascularity along the tumor edge and interstitial fluid pressure gradient at the boundary that would attenuate delivery deep into the tumor. Long circulatory half-life superimposed on the EPR effect (fenestrated or gapped ECs on capillaries or venules) leads to EC accumulation and paracellular extravasation with possibly minimal transcytosis [74]. Paracellular extravasation is most likely attributed to the defective tight junction at the tumor site. Since nanoparticles have been shown to extravasate in a size and time-dependent manner [75,76], 70-nm AuNSs can permeate into the tumor interstitial space, but only in close vicinity to tumor vessels 3 h after intravenous injection. Smaller nanoparticles or longer incubation time can further increase the tumor accumulation or extravasation depth.

## 5. AuNSs for Multi-Modality Imaging

Medical imaging modalities can be divided into structural imaging (e.g., X-ray computer tomography (CT) and magnetic resonance imaging (MRI)) and molecular imaging (e.g., positron emission tomography (PET) and optical imaging) [37]. Structural imaging and molecular imaging have their own advantages and disadvantages [77,78]. MRI and CT can be used to perform whole body scan to identify macroscopic outlines of tumors due to their low tissue attenuation and deep penetration depth. However, they cannot delineate tumor margins with high spatial resolution and sensitivity. Optical imaging can be used to detect tumors with high spatial resolution but their penetration is not deep enough for whole body scan. As a result, it is of great interest to develop a multimodal contrast agent that can combine structural imaging and molecular imaging to perform both pre-operative macroscopic and intra-operative high-resolution imaging.

**Figure 4.**
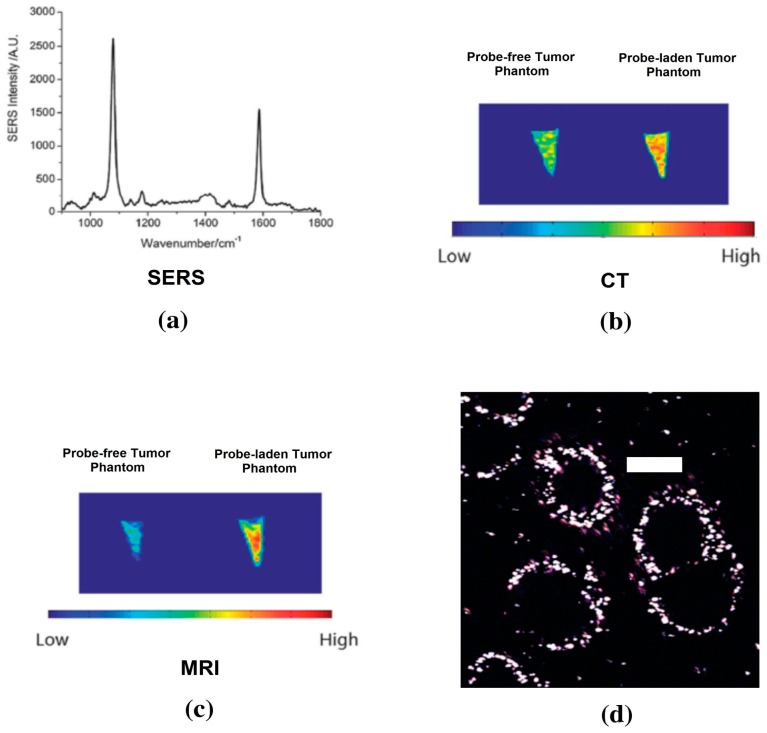
(**a**) SERS signal detected from cells with AuNSs multimodality probe; (**b**,**c**) CT and MRI imaging results for tumor phantom of probe-free tumor and probe-laden tumor cells. [37] (Adapted from Ref. [37]); (**d**) Nanostars with anti-EGFR antibody binds to SKBR-3 cell membrane. Scale bar, 20 μm. Images were acquired with two-photon microscope. The white spots are AuNSs linked with EGFR antibodies.

AuNSs provide a unique nanoplatform for multimodal imaging, which can be used for combined whole body scans with CT, MRI or PET and high resolution optical imaging with SERS and TPL [37]. The multimodal imaging nanoprobe was developed by coating pMBA-labeled AuNSs with a thin layer of silica and then functionalizing the silica surface with thiol groups. Then 1,4,7,10-tetraazacyclodedecane-1,4,7,10-tetraacetic acid (DOTA) chelators were linked to the silica surface *via* a thiol-maleimide coupling chemistry. Using the same method, polyethylene glycol (PEG) polymers were also linked to the nanoprobes in order to reduce liver/spleen uptake and improve circulation time. With DOTA chelators on AuNSs surfaces, Gd^3+^ can be linked to the multimodal nanoprobe for MRI imaging, or ^64^Cu^2+^ can be bound for PET imaging. In addition, we have also linked antibodies to the AuNSs surface to perform active targeting on cancer cells. The epidermal growth factor receptor (EGFR) antibody was linked to the AuNSs surface by the linker maleimide-PEG-NHS ester. Figure 4d shows that the AuNSs bind to the surface of SKBR-3 breast cancer cells, which overexpress EGFR on cell membranes. The developed multifunctional nanoprobe can achieve detection limits of 2 pM, 10 pM, and 100 pM for SERS, MRI and CT, respectively. The tumor phantom consisting of breast cancer cells and agar gel was used to evaluate the developed multifunctional nanoprobe. As shown in Figure 4a, the SERS spectrum detected shows clear “fingerprint” peaks of pMBA. The MRI and CT imaging results show that the tumor phantom with nanoprobe-ladened BT549 cells has higher intensity than that with nanoprobe-free BT 549 cells. In addition, for CT imaging, gold nanoparticles provide better contrast effect than traditional iodinated contrast agents because gold has a higher atomic number and k-edge value.

**Figure 5.**
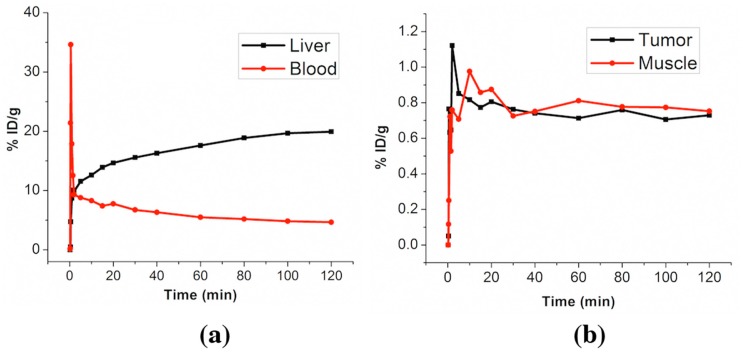
Plot of measured *in vivo* radioactivity *versus* time in liver, blood (**a**) and tumor, muscle (**b**) after tail vein injection of 100 μL radiolabeled nanoprobes in PBS solution. The radioactivity was measured on acquired PET/CT scan results.

AuNSs have also been used for *in vivo* tracking with PET imaging. The AuNSs were radiolabeled with ^64^Cu^2+^ radioisotopes by DOTA chelators linked on the nanoparticle surface. The labeling efficiency, or radiochemical yield of the reaction, was calculated to be 86.8% ± 6.6% (*n* = 3). The stability of the ^64^Cu^2+^ DOTA chelation was studied in mouse plasma before *in vivo* application. Following 24 h of incubation at 37 °C, greater than 95% of the radioactivity was retained on the nanoprobes. As shown in Figure 5a, 2-h continuous PET scan results revealed an immediate nanoprobe uptake in the liver, which is the major organ of the reticuloendothelial system (RES). The nanoprobe concentration in blood increased quickly to 34.6%ID/g at 0.5 min after IV injection. At the same time, liver uptake increased to 4.7%ID/g. At 2 min, the liver uptake increased rapidly to 9.85%ID/g while the nanoprobe in blood decreased dramatically to 12.5%ID/g. The rapid decrease of nanoprobe concentration in blood could be partially due to the filtration effect of RES organs including liver. Thereafter, the liver uptake almost doubled and the nanoprobe in blood decreased slowly to 4.65%ID/g at the end of the 2 h. We also examined the nanoprobe distribution in tumor and muscle. In the initial 2 h, tumor and muscle uptake were quite similar as shown in Figure 5b. The nanoprobe uptake increased quickly to 0.85%ID/g (tumor) and 0.75%ID/g (muscle) at the end of 2 min. Then, the nanoprobe uptake in tumor and muscle fluctuated at around 0.8%ID/g until the end of 2 h. Because muscle is not a typical organ with leaky vasculature, it is possible that only minimal EPR effect (*i.e.*, minimal extravasation) was observed in the tumor in the initial 2 h. Hence, PET signal may originate from nanoprobes in the blood vessels but not in the tumor parenchyma at this time point.

**Figure 6.**
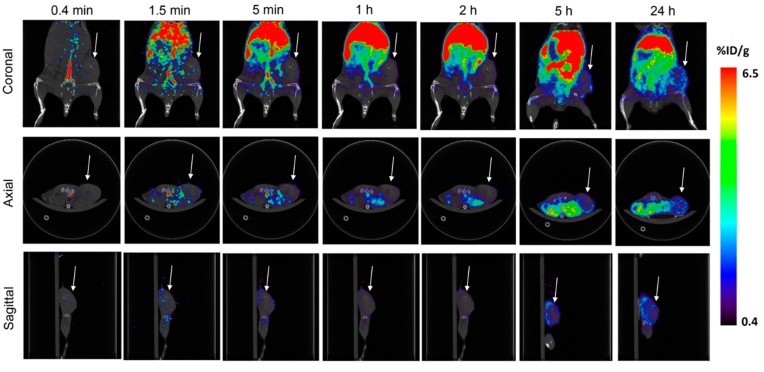
PET/CT imaging (3 orientations) of the tumor-bearing mouse following systemic nanoprobe injection. Images were obtained at 0.4, 1.5, 5 min, 1, 2, 5 and 24 h post injection, respectively. Tumor is shown under white arrow. High nanoprobe uptake in liver can be seen 1.5 min after IV injection through tail vein. Nanoprobes accumulate in tumor gradually within 24 h primarily in the tumor periphery.

Dynamic PET/CT image series within 24 h after IV injection are shown in Figure 6. Immediately after the injection (0.4 min), high signal was mostly found in major blood vessels. After 1.5 min, significant nanoprobe signal can be observed in the liver. Between 2 h and 24 h, the tumor uptake of nanoprobe increases slowly with time. Although no preferential accumulation in tumor was observed in the initial 2 h time period, the tumor-to-muscle ratio increased to 2.5:1 and 3.3:1, at the end of 5 h and 24 h, respectively. Previously, passive tumor targeting has been achieved *via* the EPR effect using nanoparticles with long circulation times [43,79]. Because of the PEGylation on the nanoprobe surface, such tumor accumulation in our study is most likely due to the EPR effect. The results demonstrate that AuNSs can be tracked *in vivo* dynamically with sensitive PET imaging.

## 6. Conclusions

Plasmonic AuNSs provide a unique multifunctional nanoplatform capable to be used as *in vivo* nanosensors and optical imaging contrast agents. AuNSs have a tunable plasmonic band in the near infrared region, where there is low tissue absorption and autofluorescence, and therefore they are quite suitable for *in vivo* applications. Our studies have demonstrated that AuNSs can be employed for high sensitivity pH sensing using SERS detection as well as for *in vivo* imaging using TPL, CT, MRI and PET detection. The superior multifunctional capability of AuNSs makes it possible to further investigate how nanoparticles can be used for cancer diagnostics and therapeutics as well as drug delivery and *in vivo* sensing for future biomedical applications.
